# Calpain-2 Compensation Promotes Angiotensin II-Induced Ascending and Abdominal Aortic Aneurysms in Calpain-1 Deficient Mice

**DOI:** 10.1371/journal.pone.0072214

**Published:** 2013-08-19

**Authors:** Venkateswaran Subramanian, Jessica J. Moorleghen, Anju Balakrishnan, Deborah A. Howatt, Athar H. Chishti, Haruhito A. Uchida

**Affiliations:** 1 Saha Cardiovascular Research Center, University of Kentucky, Lexington, Kentucky, United States of America; 2 Department of Molecular Physiology and Pharmacology, Sackler School Programs in Physiology, Pharmacology, and Microbiology, Tufts University School of Medicine, Boston, Massachusetts, United States of America; 3 Department of Medicine and Clinical Sciences, Okayama University Graduate School of Medicine, Dentistry and Pharmaceutical Sciences, Okayama, Japan; Medical University Innsbruck, Austria

## Abstract

**Background and Objective:**

Recently, we demonstrated that angiotensin II (AngII)-infusion profoundly increased both aortic protein and activity of calpains, calcium-activated cysteine proteases, in mice. In addition, pharmacological inhibition of calpain attenuated AngII-induced abdominal aortic aneurysm (AA) in mice. Recent studies have shown that AngII infusion into mice leads to aneurysmal formation localized to the ascending aorta. However, the precise functional contribution of calpain isoforms (-1 or -2) in AngII-induced abdominal AA formation is not known. Similarly, a functional role of calpain in AngII-induced ascending AA remains to be defined. Using BDA-410, an inhibitor of calpains, and calpain-1 genetic deficient mice, we examined the relative contribution of calpain isoforms in AngII-induced ascending and abdominal AA development.

**Methodology/Results:**

To investigate the relative contribution of calpain-1 and -2 in development of AngII-induced AAs, male LDLr −/− mice that were either calpain-1 +/+ or −/− were fed a saturated fat-enriched diet and infused with AngII (1,000 ng/kg/min) for 4 weeks. Calpain-1 deficiency had no significant effect on body weight or blood pressure during AngII infusion. Moreover, calpain-1 deficiency showed no discernible effects on AngII-induced ascending and abdominal AAs. Interestingly, AngII infusion induced increased expression of calpain-2 protein, thus compensating for total calpain activity in aortas of calpain-1 deficient mice. Oral administration of BDA-410, a calpain inhibitor, along with AngII-infusion significantly attenuated AngII-induced ascending and abdominal AA formation in both calpain-1 +/+ and −/− mice as compared to vehicle administered mice. Furthermore, BDA-410 administration attenuated AngII-induced aortic medial hypertrophy and macrophage accumulation. Western blot and immunostaining analyses revealed BDA-410 administration attenuated AngII-induced C-terminal fragmentation of filamin A, an actin binding cytoskeletal protein in aorta.

**Conclusion:**

Calpain-2 compensates for loss of calpain-1, and both calpain isoforms are involved in AngII-induced aortic aneurysm formation in mice.

## Introduction

Ascending and abdominal aortic aneurysms (AAs) are two major common aortic diseases that have highly distinct pathologies, and mediated by different etiologies [1]. Ascending AAs are highly associated with genetic abnormalities of connective tissue and frequently occur in younger individuals [2], [3]. Abdominal AAs show relatively weak genetic association, but are positively associated with aging, male gender and smoking [4]–[6]. Since both ascending and abdominal AAs are asymptomatic, the incidence of aortic rupture is increasing and the current therapy is restricted only to surgical repair.

Chronic infusion of angiotensin II (AngII) into hypercholesterolemic mice promotes atherosclerosis and abdominal AA formation [7]–[9]. Recently, it was recognized that AngII-infusion also leads to aneurysmal formation localized to the ascending aorta [10]. Although AngII infusion develops both ascending and abdominal AAs in the same mouse model, the underlying pathologies are clearly distinct between the two forms. AngII-induced abdominal AAs are characterized with initial small focal regions of macrophage accumulation in the aortic media [11]. AngII-induced ascending AAs are characterized with macrophage accumulation throughout the aortic circumference, predominantly on the adventitial side of the aorta [10]. Systemic deficiency of angiotensin II type 1a (AT1a) receptor completely ablates the development of AngII-induced ascending and abdominal AAs in mice [12], [13]. However, identities of key regulators and underlying mechanisms for development of these vascular pathologies remain poorly understood.

Recently, we demonstrated that pharmacological inhibition of calpain, a calcium dependent cysteine protease, using a novel and relatively specific inhibitor, BDA-410, significantly attenuated AngII-induced abdominal AA formation in LDLr −/− mice [14]. BDA-410 is an active synthetic Leu-Leu peptidomimetic with a cyclopropenone group that strongly binds to the hydrogens of the -SH residues of cysteines contained in the calpain molecule [15], [16]. BDA-410 has a potent and selective inhibitory action on calpain over other proteases. In cultured SHSY5Y cells, the inhibitory effects against specific proteases (IC_50_) are calpain 1/calpain 2 = 21 nM; papain = 400 nM; cathepsins B = 16,000 nM; thrombin >100 µM; cathepsin G, D and proteasome 20S >100 µM. The beneficial effect of calpain inhibition on abdominal AA formation was mainly associated with a reduction in medial macrophage accumulation and inflammation [14]. The two major isoforms of the calpain family, calpain-1 and calpain-2, are ubiquitously expressed along with their endogenous inhibitor, calpastatin, whereas other isoforms such as calpain-3, calpain-8, and calpain-9 show tissue-specific expression [17]. However, the precise functional contribution of either calpain isoform, in the development of AngII-induced abdominal AA formation remains unknown. Similarly, a functional role of calpain activity in the development of AngII-induced ascending AA remains to be defined. Using the pharmacological inhibitor of calpains, BDA-410, and calpain-1 genetic deficient mice, we examined the relative contribution of calpain-1 and -2 isoforms in development of AngII-induced ascending and abdominal AAs. Our findings demonstrate a functional role of both calpain isoforms in development of AngII-induced vascular pathology.

## Methods

### Ethics Statement

This study followed the recommendations of The Guide for the Care and Use of Laboratory Animals (National Institutes of Health). All mouse studies were performed with approval by the University of Kentucky’s Institutional Animal Care and Use Committee (Protocol # 2011-0907). The mice were observed daily for any signs of distress and weighed weekly to monitor health. Pump implantation was conducted using isoflurane inhalation anesthesia, and termination was performed with overdose of ketamine/xylazine.

### Mice

LDL receptor −/− (stock # 002207) mice were purchased from The Jackson Laboratory (Bar Harbor, ME). Calpain-1 −/− mice on a C57BL/6 background were originally generated in the laboratory of Dr. Athar Chishti [18], [19]. LDL receptor −/− and calpain-1 −/− mice were backcrossed 10 times into the C57BL/6 background. To generate study mice in an LDL receptor −/− background, calpain-1 −/− males were mated to LDL receptor −/− females, and their offspring were bred to generate calpain-1+/− males and females in the LDL receptor −/− genotype. Subsequent breeding generated relevant littermate controls of calpain-1 +/+ x LDL receptor −/− and calpain-1 −/− x LDL receptor −/− mice. Age-matched male littermates (8–10 weeks old) were used for the present study. Mice were maintained in a barrier facility and fed normal mouse laboratory diet. All study procedures were approved by the University of Kentucky Institutional Animal Care and Use Committee.

### Mouse Genotyping

Mouse genotypes were confirmed by PCR. DNA was isolated from tail snips using a DNeasy tissue kit (Cat# AS1120, Promega, Madison, WI). Calpain-1 genotyping was performed using the following primers: 5'-TGCACTCTAGTTCTGAGG CT-3', 5'-AGAGTGCACGAACACCAGCTT-3', and 5'-TTAAGGGCCAGCTCATTCCT-3'. PCR of wild-type and disrupted alleles generated amplicons of 615 bp and 415 bp, respectively. A representative gel of calpain-1 +/+ and −/− amplicons is shown in the Figure S1 in File S1. LDL receptor genotyping was performed as described previously [20].

### Diet

To induce hypercholesterolemia, mice were fed a diet supplemented with saturated fat (21% wt/wt milk fat; TD.88137, Harlan Teklad, Indianapolis, IN) for 2–5 weeks.

### Calpain Inhibitor BDA-410 Administration

BDA-410 was a kind gift from the Mitsubishi Tanabe Pharma Corporation, Osaka, Japan. The BDA-410 compound was pulverized and suspended in 1% Tween 80 diluted in saline and administered daily for 2 or 5 weeks by gavage at a dose of 30 mg/kg/day [15].

### AngII Infusion

After an initial week of high-fat diet feeding, mice were implanted with Alzet osmotic minipumps (model 1004 or 2004, Durect Corporation, Cupertino, CA), subcutaneously into the right flank, and infused with AngII (1,000 ng/kg/min, Bachem, Torrance, CA) continuously for a period of 7,14 or 28 days, as described previously [7]. Mice were maintained on high fat-enriched diet throughout the study. For calpain inhibition studies, along with high fat diet and AngII infusion, mice were gavaged daily with the calpain inhibitor, BDA-410.

### Blood Pressure Measurement

Systolic blood pressure (SBP) was measured noninvasively on conscious mice by volume pressure recording of the tail using a computerized tail cuff blood pressure system (Kent Scientific Corp, Torrington, CT) [21]. SBP was measured on 5 consecutive days prior to pump implantation, and during the last 5 days of the AngII infusion.

### Measurement of Plasma Components

Plasma cholesterol concentrations were measured using a commercially available enzymatic kit (Wako Chemicals, Richmond, VA) and lipoprotein cholesterol distribution was determined as described previously [7], [20].

### Ultrasound Imaging of Abdominal AA

Luminal dilation of the abdominal aorta was measured by a high frequency ultrasound imaging system (Vevo 2100, Visual Sonics, Toronto, Canada) using a MS400 MicroScan™ transducer with a resolution frequency of 18–38 MHz [22]. Mice were anesthetized and restrained in a supine position to acquire ultrasonic images. Short axis scans of abdominal aortas were performed from the left renal arterial branch level to the suprarenal region [22]. Images of abdominal aortas were acquired and measured to determine maximal diameter in the suprarenal region of the abdominal aorta. Aortic images were acquired at day 0 and 28 of AngII-infusion.

### Quantification of Atherosclerosis and Ascending and Abdominal AAs

Atherosclerosis was quantified on aortic arches as lesion area on the intimal surface by en face analysis as described previously [23], [24]. For aneurysm measurements, after saline perfusion through the left ventricle of the heart, aortas were removed from the origin to iliac bifurcation, and placed in formalin (10% wt/vol) overnight. Adventitial fats were cleaned from the aortas. Abdominal AAs were quantified *ex vivo* by measuring the maximum external width of the suprarenal abdominal aortic diameter using computerized morphometry (Image-Pro Cybernetics, Bethesda, MD) as described previously [25]. For ascending AA measurement, aortas were cut open longitudinally from the inner arch curvature to the iliac bifurcation, as well as from the outer curvature to the subclavian branch. Aortas were pinned and photographed using a Nikon Digital Camera (DXM1200). Intimal areas of ascending aortas were measured from the ascending aorta to the subclavian branch using Image-Pro Plus software [10], [12].

### Tissue Histology and Immunostaining

Ascending and abdominal aortas were placed in OCT and sectioned (10 µm thickness/section) in sets of 10 slides serially with 9 sections/slide by a cryostat [12]. One of the slides was stained with Movat’s pentachrome (Polyscientific) and measurements were performed to determine medial thickness and elastin breaks. To quantify medial thicknesses, every section from each slide was measured perpendicular from internal to external elastic lamina [12]. Immunohistochemical staining was performed on AA sections to detect calpain-1, calpain-2, macrophages, smooth muscle cells, fibroblasts and endothelial cells. Calpain-1 and -2 immunostaining were performed using the rabbit anti-mouse calpain-1 and -2 (aminoterminal end domain-I, 3 µg/ml, catalog Nos. RP1-Calpain-1 and RP-2 Calpain-2; Triple Point biologics, Forest Grove, OR). The following reagents were used to detect specific cell types: rat anti-mouse CD68 (1∶200, catalog No. MCA1957; Serotec, Raleigh, NC) and rabbit antisera (1∶1000, catalog No. AI-AD31240; Accurate Chemical, Westbury, NY) for macrophages; rabbit anti-mouse α-smooth muscle actin (2 µg/ml, catalog No: ab5694; Abcam, Cambridge, MA) for smooth muscle cells; rat anti-mouse reticular fibroblasts and reticular fibers (1 µg/ml, catalog No: ab51824; Abcam, Cambridge, MA) for fibroblasts. In addition we also attempted to immunostain for endothelium using rat anti-mouse platelet endothelial cell adhesion molecule (PECAM)-1 (CD31, 1∶1000; catalog No. 553371; BD Pharmingen, San Jose, CA ) and rabbit anti-mouse CD31 (1∶100; catalog No. RB-10333-P1; Lab Vision Corporation, Fremont, CA) and for spectrin using rabbit anti-mouse spectrin (1 µg/ml; catalog No. A301-249A, Bethyl Laboratories, Montgomery, TX). Immunostaining was performed on formalin-fixed frozen sections, with appropriate negative controls, as described previously [20], [26].

### Western Blot Analyses

Whole aortic tissue lysates were extracted in radio immunoprecipitation assay (RIPA) lysis buffer and protein content was measured using the Bradford assay (Bio-Rad, Hercules, CA). Protein extracts (30 µg) were resolved by SDS-PAGE (6 or 7.5% wt/vol) and transferred electrophoretically to PVDF membranes. After blocking with non-dry fat milk (5% wt/vol), membranes were probed with primary antibodies. The following antibodies against calpain-1 domain IV (catalog No: ab39170), calpain-2 (catalog No: ab39165) and filamin A (catalog No: ab76289) were purchased from Abcam, Cambridge, MA. Spectrin (catalog No: MAB1622) antibody was purchased from Chemicon-EMD Millipore, Billerica, MA. Calpastatin (catalog No. PA5-17068) antibody was purchased from Pierce, Rockford, IL. β-actin (catalog No: A5441) antibody was purchased from Sigma-Aldrich, St. Louis, MO. Membranes were incubated with appropriate HRP-labeled secondary antibodies, immune complexes were visualized by chemiluminescence (Pierce, Rockford, IL) and quantified using a Kodak Imager.

### Calpain Activity Assay

Calpain enzyme activity was measured in aortic tissue lysates fluorimetrically using a commercially-available activity assay kit (Calpain, catalog No: K240-100; BioVision, Mountain View, CA). Aortic protein extracts (20 µg) were incubated with fluorogenic (4-trifluoromethyl coumarin labeled) calpain substrate for 60 min at 37°C. Mean fluorescence signals were measured using a microplate fluorescent plate reader (Spectramax M2; Molecular Devices, Sunnyvale, CA) as per manufacturer’s instructions.

### Statistical Analyses

Data are represented as mean ± SEM. Statistical analyses were performed using SigmaPlot 12.0 (SYSTAT Software Inc., San Jose, CA, USA). Repeated measurement data were analyzed with SAS fitting a linear mixed model expressing the temporal trend in systolic blood pressure as a quadratic polynomial in time for each treatment. Ultrasound measurements of abdominal aortic diameter measured on day 0 and day 28 were analyzed using one or two way repeated measures ANOVA. Student’s *t* test or Mann-Whitney Rank Sum test was performed as appropriate for two-group comparisons. Two way ANOVA with Holm-Sidak post hoc analysis was performed for multiple-group and multiple-manipulation analysis. Values of *P*<0.05 were considered to be statistically significant.

## Results

### Calpains are Present in AngII-induced Abdominal AAs

**Figure 1:**
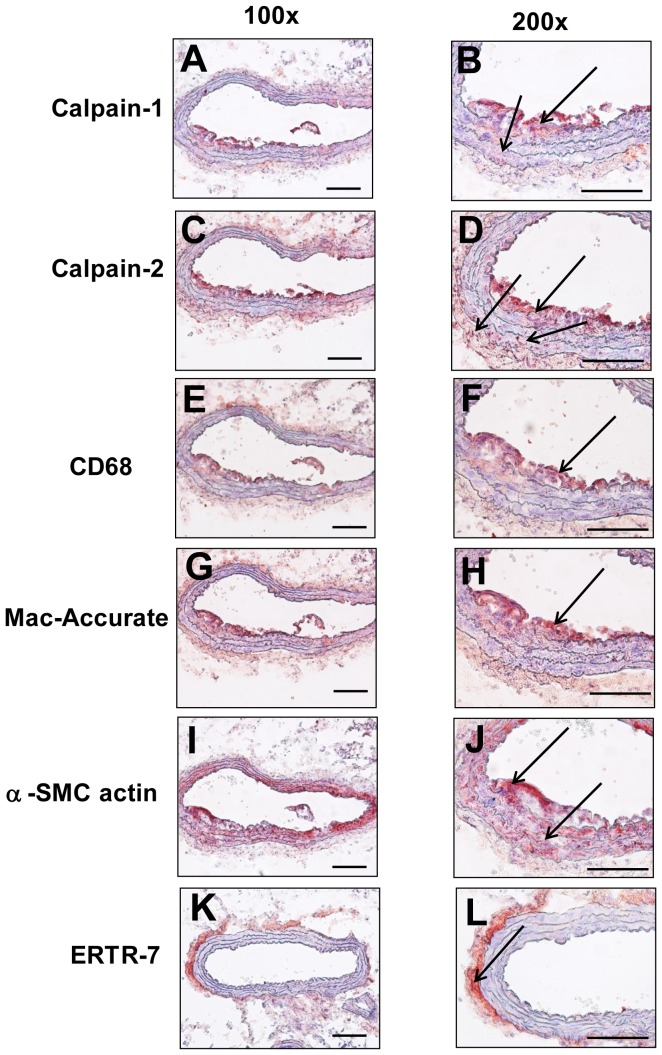
Calpains are present in AngII-induced abdominal AAs. Cross-sections from the abdominal aorta of LDLr −/− mice infused with AngII for 28 days were immunostained with anti-mouse calpain-1/-2 (**A–D**) or with cell-specific markers. Immunostaining with rat anti-mouse CD68 or rabbit anti-sera for mouse macrophages (**E–H**), rabbit α-SMC actin for SMCs (**I,J**), and ERTR-7 for fibroblasts (**K,L**). Arrow indicates positive red staining. Scale bars correspond to 50 µm. A,C,E,G,I and K = 100×; B,D,F,H,J and L = 200×.

Immunostaining using calpain-1, -2 and cell specific antibodies were performed on AngII-induced abdominal AA sections to examine the distribution and localization of calpains in abdominal AAs. Positive immunostaining for calpain-1 and -2 (Fig. 1A–D) was most pronounced in regions containing macrophages (Fig. 1E–H). Diffused immunostaining was also observed in the aortic media (Fig. 1I,J) and adventitia (Fig. 1K,L).

### Calpain-1 Deficiency had No Effect on AngII-induced Abdominal AAs

**Figure 2:**
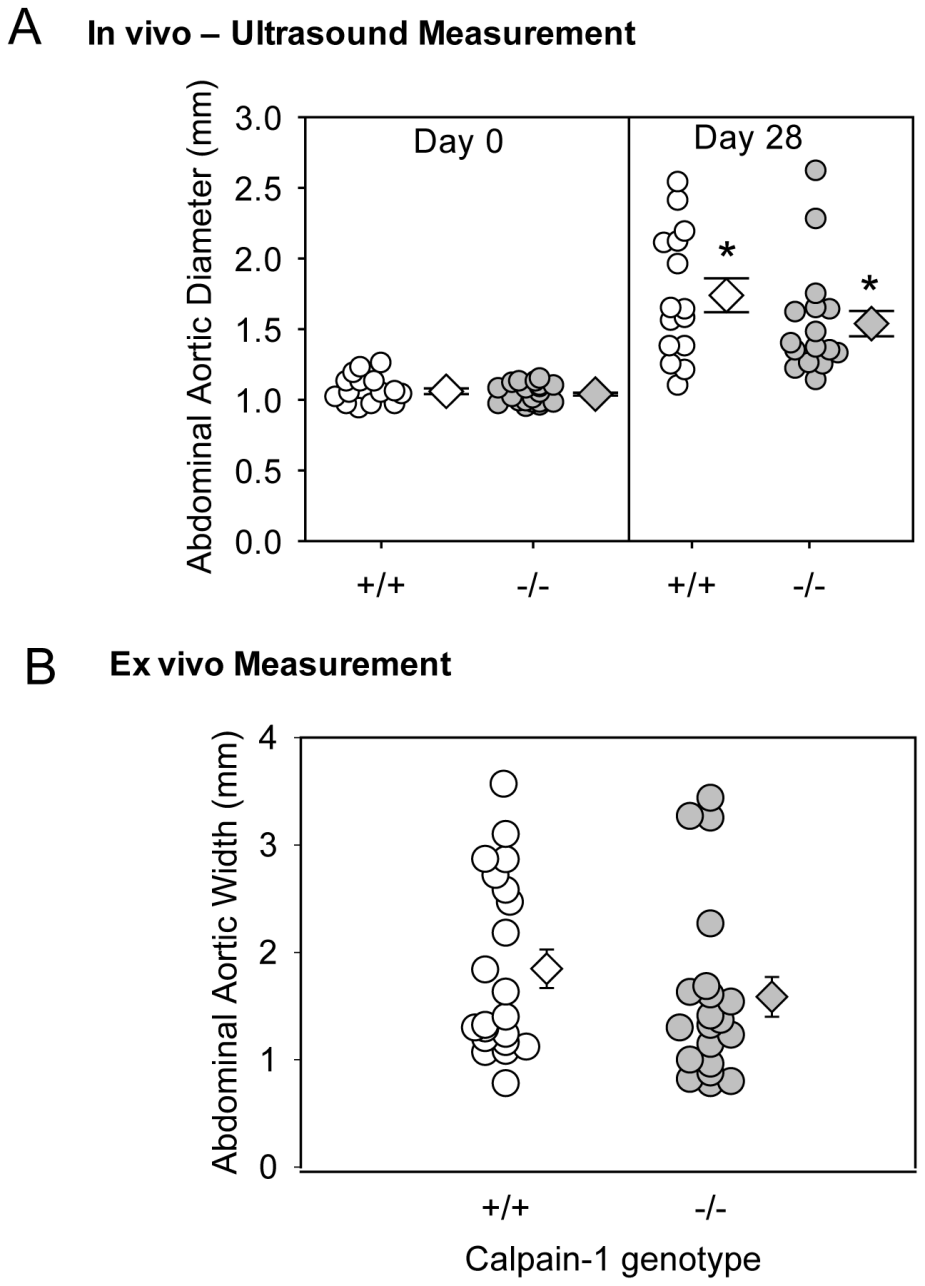
Calpain-1 deficiency had no effect on AngII-induced abdominal AA formation. **A**. Ultrasonic measurements of abdominal aortic diameters were measured on day 0 and after 28 days of AngII-infusion (n = 15–20). **B**. Measurements of maximal external width of abdominal aortas on day 28 (n = 20–21). Open circles (calpain-1 +/+) and gray circles (calpain-1 −/−) represent individual mice, diamonds represent means, and bars are SEMs. Statistical analyses were performed using repeated measures ANOVA with a Holm-Sidak multiple comparison post-hoc test (**A**), or nonparametric Mann-Whitney Rank sum test (**B**). * denotes *P*<0.05 when comparing saline versus AngII-infused mice.

To determine the role of calpain-1 in AngII-induced abdominal AA formation, male LDL receptor −/− mice with either calpain-1 +/+ or −/− genotype were fed a high fat-enriched diet, and infused with AngII (1,000 ng/kg/min) for 28 days. AngII infusion significantly increased SBP in both groups (*P*<0.001; Table 1). Calpain-1 deficiency or AngII-infusion had no effect on body weight, plasma total cholesterol concentrations (Table 1), or lipoprotein cholesterol distribution (Figure S2 in File S1). AngII-infusion significantly increased luminal dilation of abdominal aortas as measured by ultrasound on day 28 compared to day 0 in both animal groups (*P*<0.05; Fig. 2A). However, there was no significant difference between the two groups at 28 days. In addition, calpain-1 deficiency did not influence the formation of AngII-induced abdominal AA as measured by *ex vivo* external aortic width (Fig. 2B). Examples of ultrasound photographs and *ex vivo* images of abdominal aortas are shown in the Figures S3 A and B. Moreover, calpain-1 deficiency did not exert any significant effect on AngII-induced atherosclerosis. Atherosclerosis lesion size was quantified on the intimal surface of the aortic arch (Figure S4 in File S1).

**Table 1:** Effects of calpain-1 deficiency in male LDL receptor −/− mice infused with AngII.

Groups	Calpain-1 +/+	Calpain-1 −/−
N	20	21
Body Weight (g)	30±1	29±1
Plasma Cholesterol Concentrations(mg/dL)	1589±39	1531±70
Systolic BP Pre-infusion (mmHg)	122±4	123±6
Systolic BP Post-infusion (mmHg)	156±5*	153±6*

Values are represented as means ± SEMs. Body weights and plasma cholesterol concentrations were determined at termination. One way repeated measures ANOVA was used to analyze systolic blood pressures.

* Denotes *P*<0.001 systolic BP post-infusion vs pre-infusion, by one-way repeated measures ANOVA. There were no significant differences between the calpain-1 genotypes for body weight, plasma cholesterol and systolic BP.

Histological features of aortic medial breaks in the abdominal aortas were examined by Movat’s Pentachrome staining (Figures S5 A-D in File S1). Furthermore, immunostaining using CD68 antibodies was performed to examine macrophage accumulation. AngII infusion showed occurrence of focal elastin disruption in abdominal aortas that was associated with accumulation of CD68+ macrophages (Figures S5 E-H in File S1). However, the effects were comparable between calpain-1 +/+ and −/− mice.

### Calpain-1 Deficiency had No Effect on AngII-induced Ascending AAs

**Figure 3:**
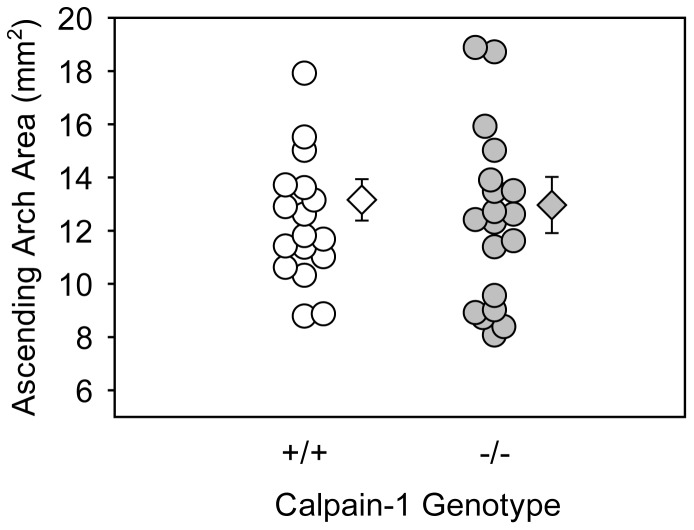
Calpain-1 deficiency had no effect on AngII-induced ascending AA formation. Measurements of ascending aortic arch intimal area after 28 days of AngII-infusion (n = 17–19). Open circles (calpain-1 +/+) and gray circles (calpain-1 −/−) represent individual mice, diamonds represent means, and bars are SEMs. Statistical analyses were performed using nonparametric Mann-Whitney Rank sum test.

Recently, it has been reported that chronic AngII infusion also induces ascending AAs. [10] Here, we examined the effect of calpain-1 deficiency on AngII-induced ascending AAs. Dilation of the ascending aortas was determined by measuring intimal area of the ascending aorta. No significant differences were observed between the two different genotypes (Fig. 3). Examples of ex vivo images of ascending arch area are shown in the Figure S3 C in File S1.

Histology (Movat’s Pentachrome) and CD68 immunostaining on ascending aortic sections showed occurrence of focal elastin disruption (Figures S6 A-D in File S1) associated with CD68+ macrophage accumulation (Figures S6 E-H in File S1). However, the effects were comparable between the two groups of mice.

### AngII Infusion Increased Calpain-2 Protein Abundance and Compensated Aortic Calpain Activity in Calpain-1 Deficient Mice

**Figure 4:**
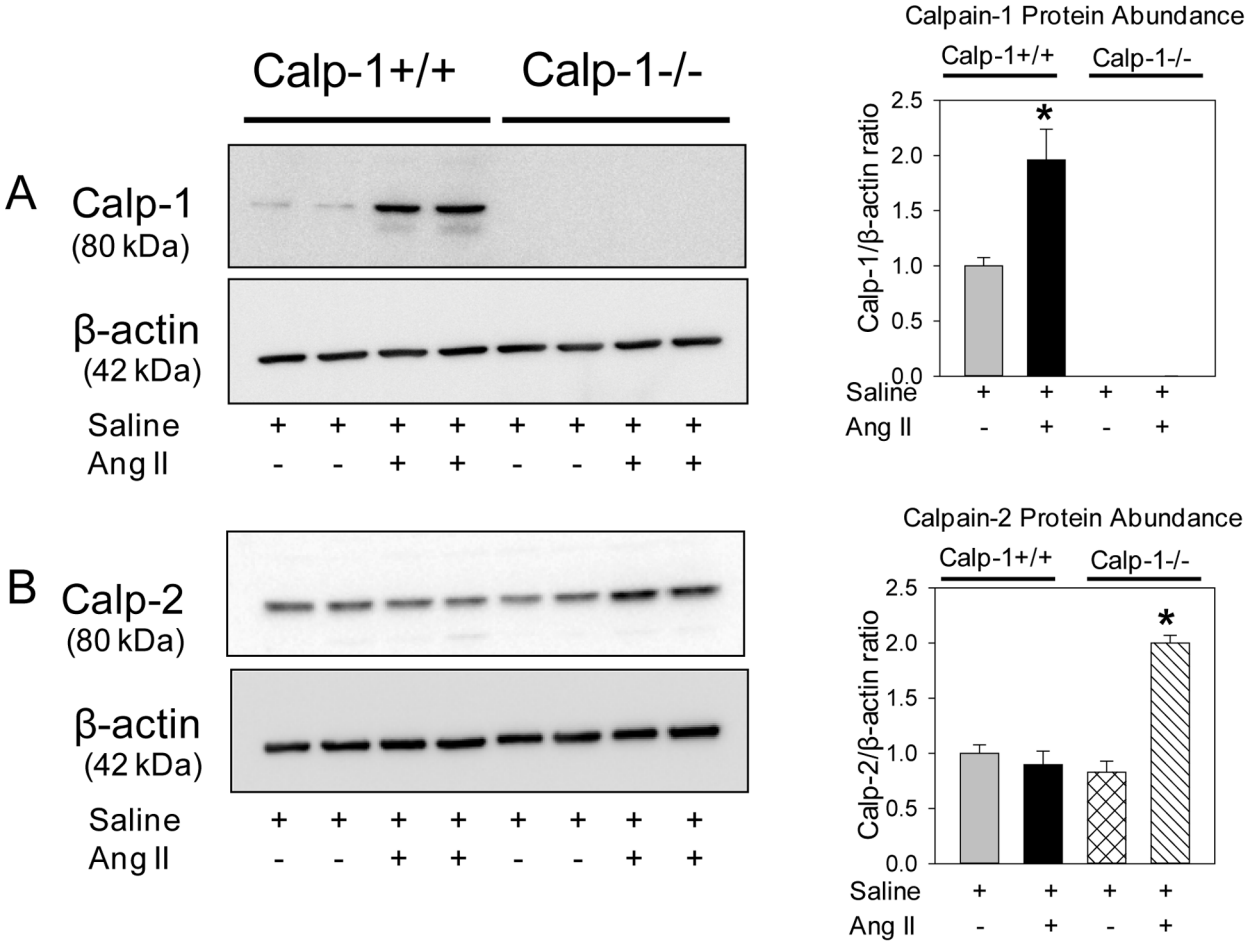
Elevated calpain protein in AngII infused calpain-1 +/+ and calpain-1 −/− aortas. Calpain-1 (**A**) and calpain-2 (**B**) proteins were detected by Western blotting in tissue extracts from aortas in calpain-1 +/+ x LDL receptor −/− and calpain-1 −/− x −/− LDL receptor −/− mice infused with either saline or AngII for 7 days. β-actin was shown as loading control. Calpain-1 (**A**) and calpain-2 (**B**) protein abundance was quantified by image analysis. Images are representative out of 4 independent experiments. Results are represented as means ± SEMs; Statistical analyses were performed using Students t test (**A**) or two-way ANOVA with a Holm-Sidak multiple comparison post-hoc test (**B**). * represent significance of *P*<0.05.

**Figure 5:**
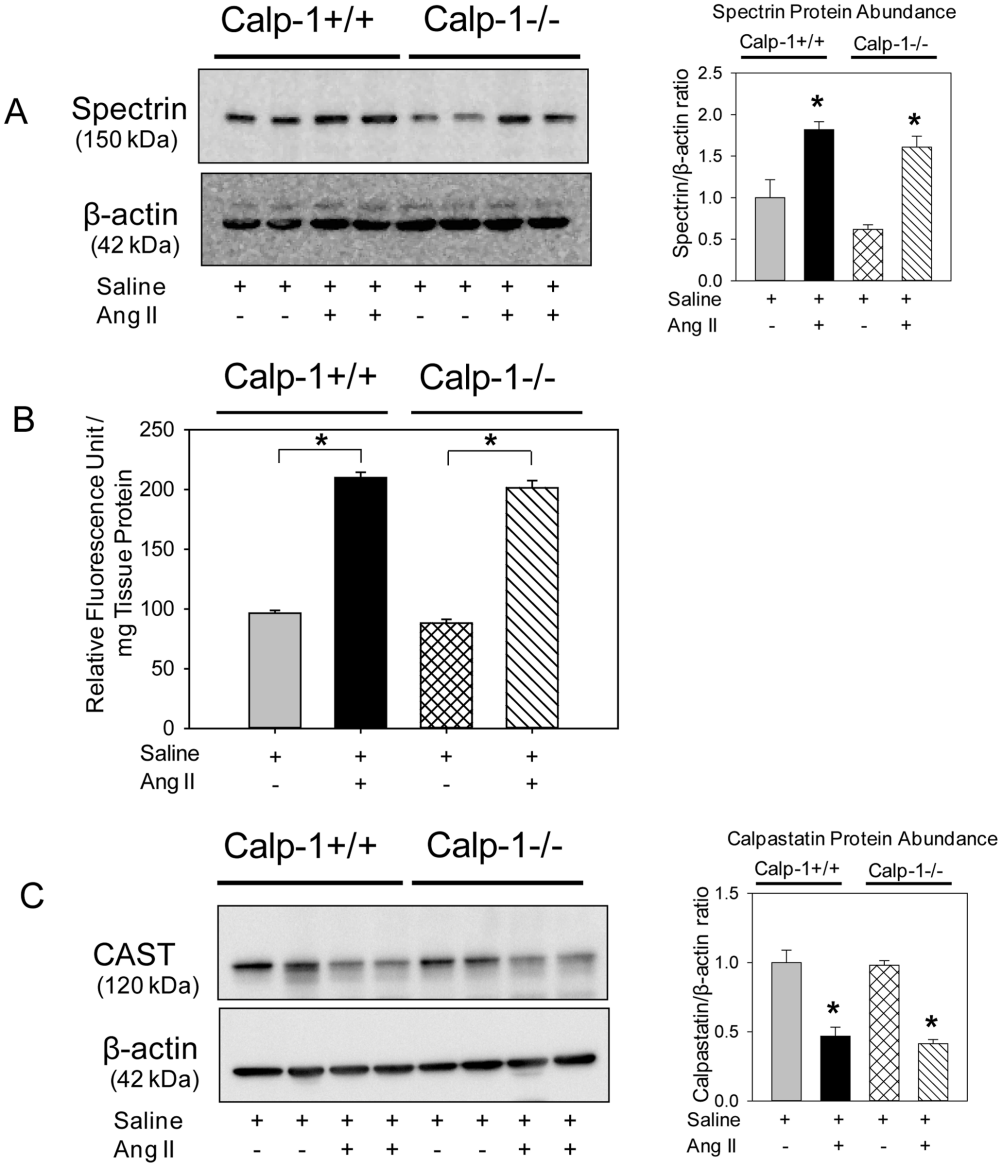
Elevated calpain activity in AngII infused calpain-1 +/+ and calpain-1 −/− aortas. Spectrin breakdown products (**A**) were detected by Western blotting in tissue extracts from aortas in calpain-1 +/+ x LDL receptor −/− and calpain-1 −/− x −/− LDL receptor −/− mice infused with either saline or AngII for 7 days as a measure of calpain activity. β-actin was shown as loading control. Calpain activity (**B**) was also measured by a fluorimetric assay in aortic tissue extracts from saline and AngII infused calpain-1 +/+ and −/− mice (n = 4). Calpastatin (**C**) was detected by Western blotting in aortic tissue extracts. Spectrin (**A**) and calpastatin (**C**) protein abundance was quantified by image analysis. Images are representative out of 4 independent experiments. Results are represented as means ± SEMs; Statistical analyses were performed using two-way ANOVA with a Holm-Sidak multiple comparison post-hoc test (**A**–**C**). * and horizontal bars represent significance of *P*<0.05.

Since calpain-1 deficiency had no effect on AngII-induced ascending and abdominal AAs, we investigated the possibility whether loss of calpain-1 may be compensated by an increase of calpain-2 during AngII infusion. Male LDL receptor −/− mice that were either calpain-1 +/+ or −/− genotype were fed a high fat-enriched diet and infused with either saline or AngII (1000 ng/kg per minute) for 7 days. Aortas were minced and protein content was extracted using a lysis buffer. Western blotting using a calpain-1 specific antibody demonstrated that AngII-infusion increased calpain-1 protein abundance in calpain-1 +/+ mice and none in calpain-1 −/− aortas, further phenotypically confirming the deficient genotype (Fig. 4A). Similarly, Western blotting using a calpain-2 specific antibody showed that basal level of calpain-2 was similar between calpain-1 +/+ and −/− mice infused with saline. Interestingly, AngII infusion significantly increased calpain-2 protein abundance in aortas of calpain-1 −/− mice, not in calpain-1 +/+ mice (Fig. 4B). To further confirm whether increased calpain-2 protein compensates for total calpain activity in aortas of calpain-1 deficient mice, breakdown product of spectrin, the major substrate of calpain, [27], [28] was analyzed by Western blot. Western blotting using a spectrin specific antibody demonstrated slightly reduced breakdown of spectrin in aortas from calpain-1 −/− mice infused with saline. However, AngII infusion showed a comparable significant increase in spectrin breakdown product in both calpain-1 +/+ and −/− mice (Fig. 5A). The increased calpain activity was further confirmed by measuring calpain activity in aortic tissue extracts using a fluorescent substrate of calpain, Ac-LLY-AFC. AngII infusion showed a comparable 2-fold increase in calpain activity in both calpain-1 +/+ and −/− mice compared to the saline group (Fig. 5B). These results demonstrate that increased calpain-2 protein compensates for total calpain activity in calpain-1 deficient mice during AngII infusion. In addition, Western blot analyses using antibodies against calpastatin, an endogenous inhibitor of calpains, [29], [30] revealed that AngII infusion significantly suppressed calpastatin protein abundance in aortas of both calpain-1 +/+ and −/− mice (Fig. 5C).

### Administration of a Calpain Inhibitor, BDA-410, Attenuated AngII-induced Abdominal AA Formation in both Wild Type and Calpain-1 Deficient Mice

**Figure 6:**
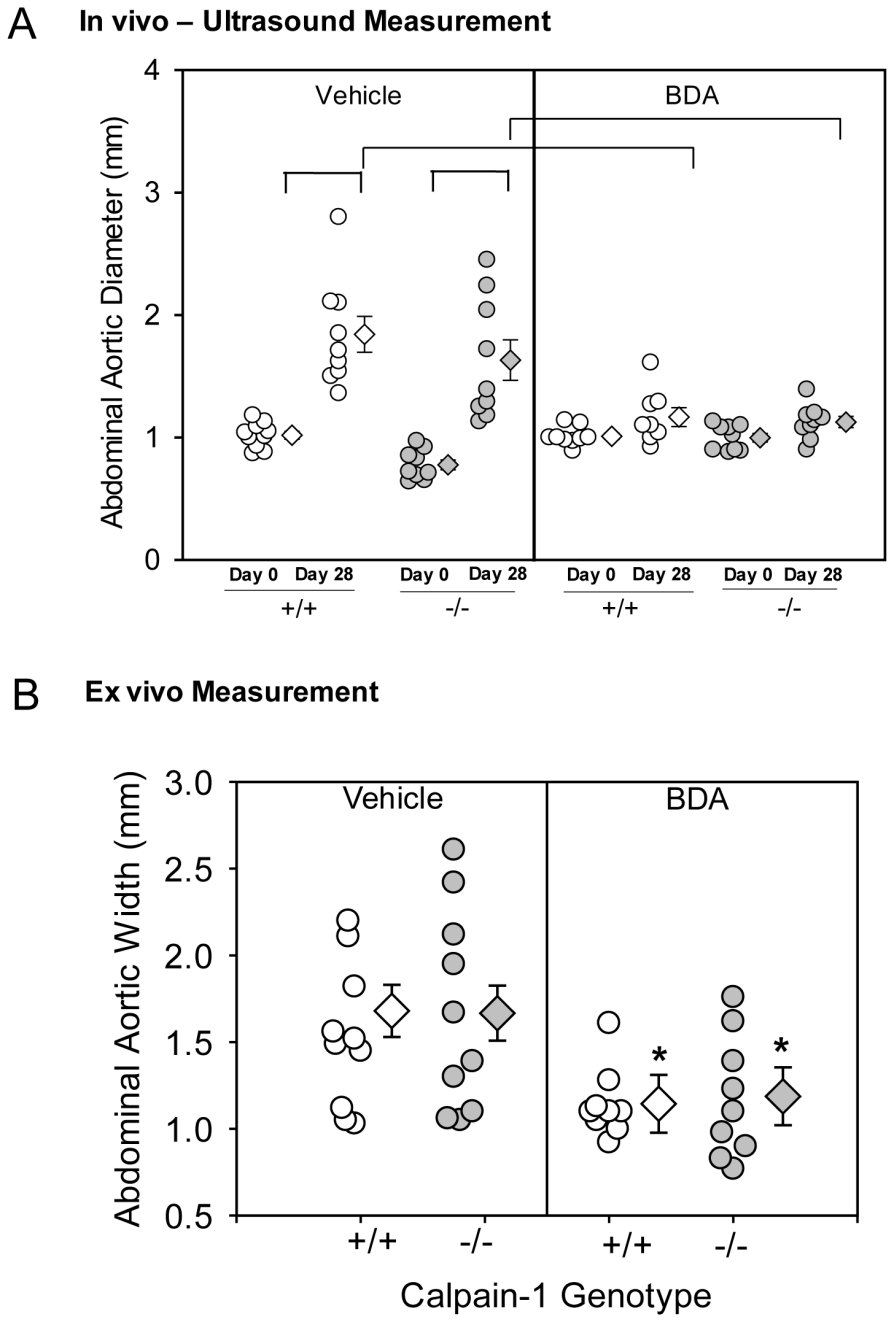
BDA-410 administration attenuated AngII-induced abdominal aortic luminal dilation and expansion in calpain-1 deficient mice. **A**. Ultrasonic measurements of abdominal aortic diameters of calpain-1 +/+ and −/− mice administered with either vehicle or BDA-410 were measured on day 0 and after 28 days of AngII-infusion (n = 10–12). **B**. Measurements of maximal external width of abdominal aortas on day 28 (n = 10–12). Open circles (calpain-1 +/+) and gray circles (calpain-1 −/−) represent individual mice, diamonds represent means, and bars are SEMs. Statistical analyses were performed using repeated measures ANOVA (**A**), or Two way ANOVA with a Holm-Sidak multiple comparison post-hoc test (**B**). Horizontal bars represent significance of *P*<0.05 when comparing day 0 and day 28. * denotes *P*<0.05 when comparing vehicle versus BDA-410.

To determine whether compensatory increase of calpain-2 contributes to development of AngII-induced AAs in calpain-1 deficient mice, male LDL receptor −/− mice that were either calpain-1 +/+ or −/− fed with a high fat enriched diet were infused with either saline or AngII (1000 ng/kg per minute) for 28 days. The calpain inhibitor, BDA-410, was administered at a dose of 30 mg/kg per day by oral gavage 1 week before infusion and throughout the subsequent 28 days [14]. Administration of BDA-410 had no effect on body weight and plasma total cholesterol concentrations (Table 2). AngII infusion significantly increased luminal dilation of abdominal aortas in vehicle-administered calpain-1 +/+ and −/− groups, as measured by ultrasound on day 28 (*P*<0.05; Fig. 6A). In contrast, administration of BDA-410 significantly attenuated AngII-induced luminal dilation of abdominal aortas in both calpain-1 +/+ and −/− groups (*P*<0.05; Fig. 4A). Inhibition of calpain activity by BDA-410 significantly attenuated the formation (*P*<0.05; Fig. 6B) of AngII-induced abdominal AA in both calpain-1 +/+ and −/− mice.

**Table 2:** Effects of BDA-410 administration on calpain-1 deficiency in male LDL receptor −/− mice infused with AngII.

Groups	Calpain-1 +/+		Calpain-1 −/−	
Drug	Vehicle	BDA-410	Vehicle	BDA-410
N	13	12	12	12
Body Weight (g)	32±1	33±1	32±1	30±1
Plasma Cholesterol(mg/dL)	1434±84	1647±96	1661±99	1558±65

Values are represented as means ± SEMs. Body weights and plasma cholesterol concentrations were determined at termination. There were no significant differences between the calpain-1 genotypes for body weight and plasma cholesterol concentrations.

**Figure 7:**
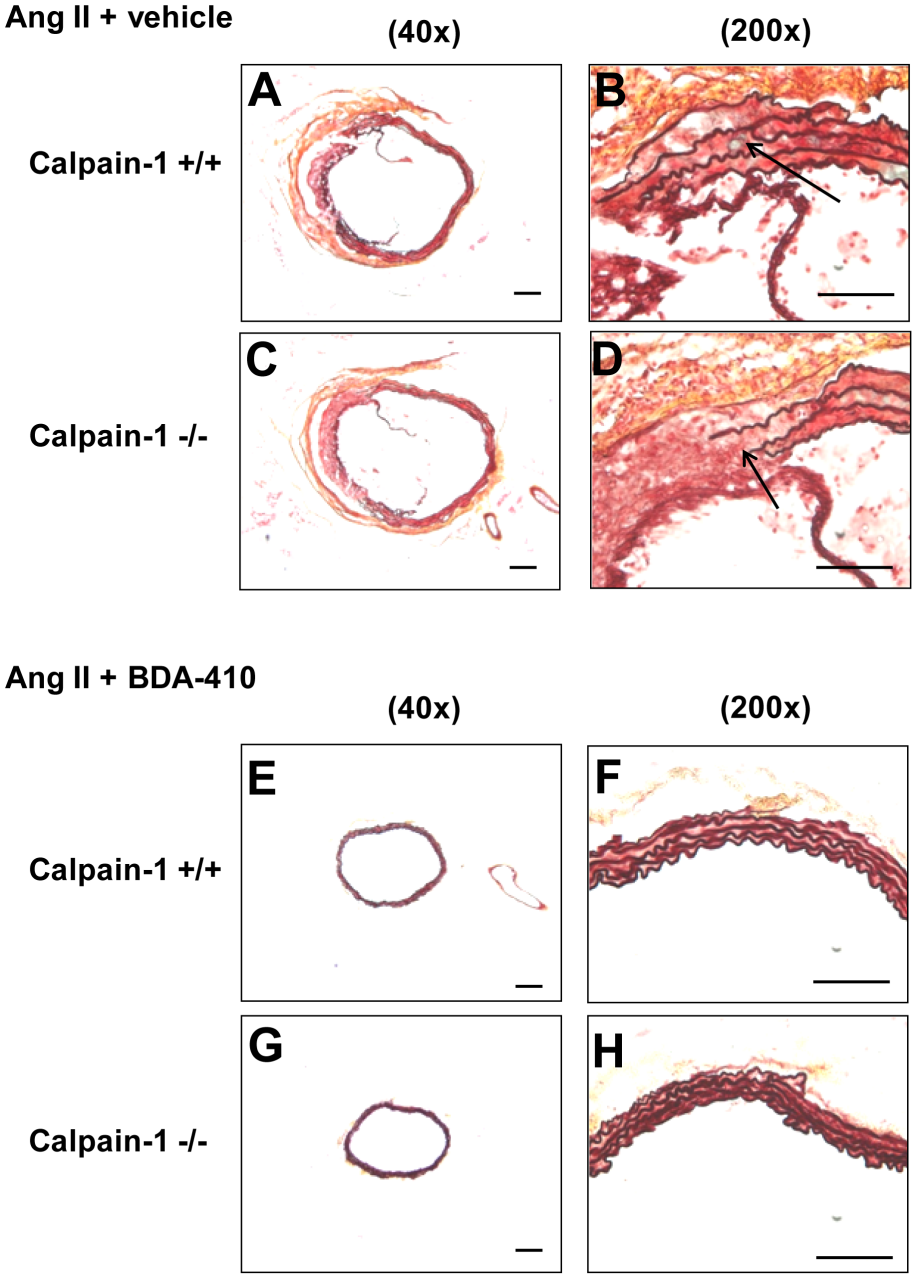
BDA-410 administration suppressed abdominal aortic medial disruption in calpain-1 +/+ and −/− mice. Representative suprarenal aortic tissue-sections from AngII+vehicle (**A–D**) and AngII+BDA-410 (**E–H)** administered calpain-1 +/+ and calpain-1 −/− mice stained with Movat’s pentachrome. Arrow indicates medial break. Scale bars correspond to 50 µm. A,C,E and G = 40×; B,D,F and H = 200×.

**Figure 8:**
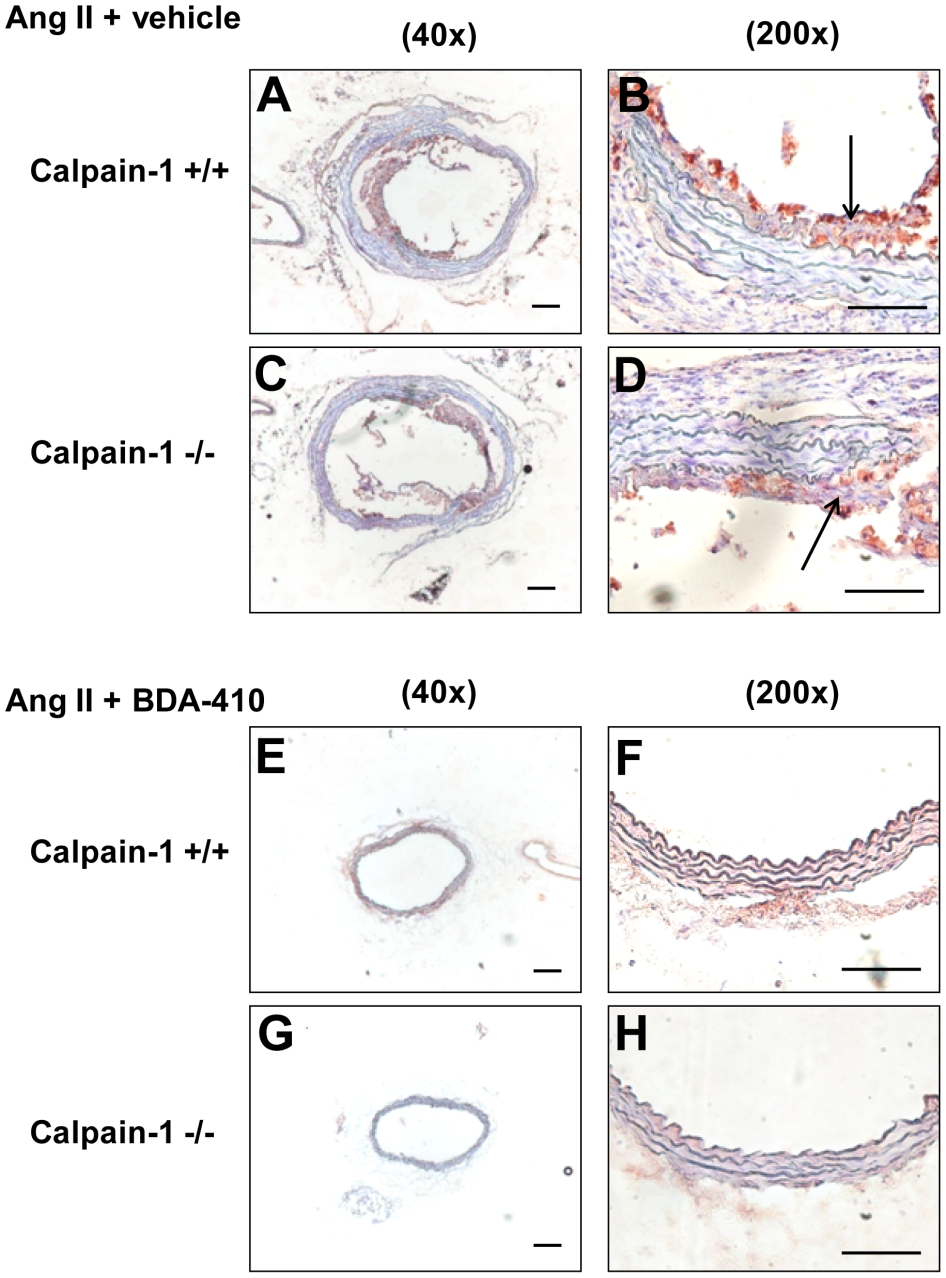
BDA-410 administration reduced macrophage accumulation in abdominal aortas of calpain-1 +/+ and −/− mice. Representative suprarenal aortic tissue-sections from AngII+vehicle (**A–D**) and AngII+BDA-410 (**E–H)** administered calpain-1 +/+ and calpain-1 −/− mice immunostained for CD68 (**E–H)**. CD68+ cells stain red. Arrow indicates positive staining with CD68. Scale bars correspond to 50 µm. A,C,E and G = 40×; B,D,F and H = 200×.

Histology and immunostaining of abdominal aortas using Movat’s Pentachrome staining and anti-CD68 antibodies respectively revealed occurrence of focal elastin layer disruption (Fig. 7 A–D) associated with the accumulation of CD68+ macrophages (Fig. 8 A–D) with AngII infusion in both calpain-1 +/+ and −/− groups. However, administration of calpain inhibitor, BDA-410, along with AngII infusion preserved the medial elastin layer (Fig.7 E–H), and attenuated macrophage accumulation (Fig. 8 E–H) in the abdominal aorta of both groups.

### Administration of Calpain Inhibitor, BDA-410, Attenuated AngII-induced Ascending AA Formation in both Wild Type and Calpain-1 Deficient Mice

**Figure 9:**
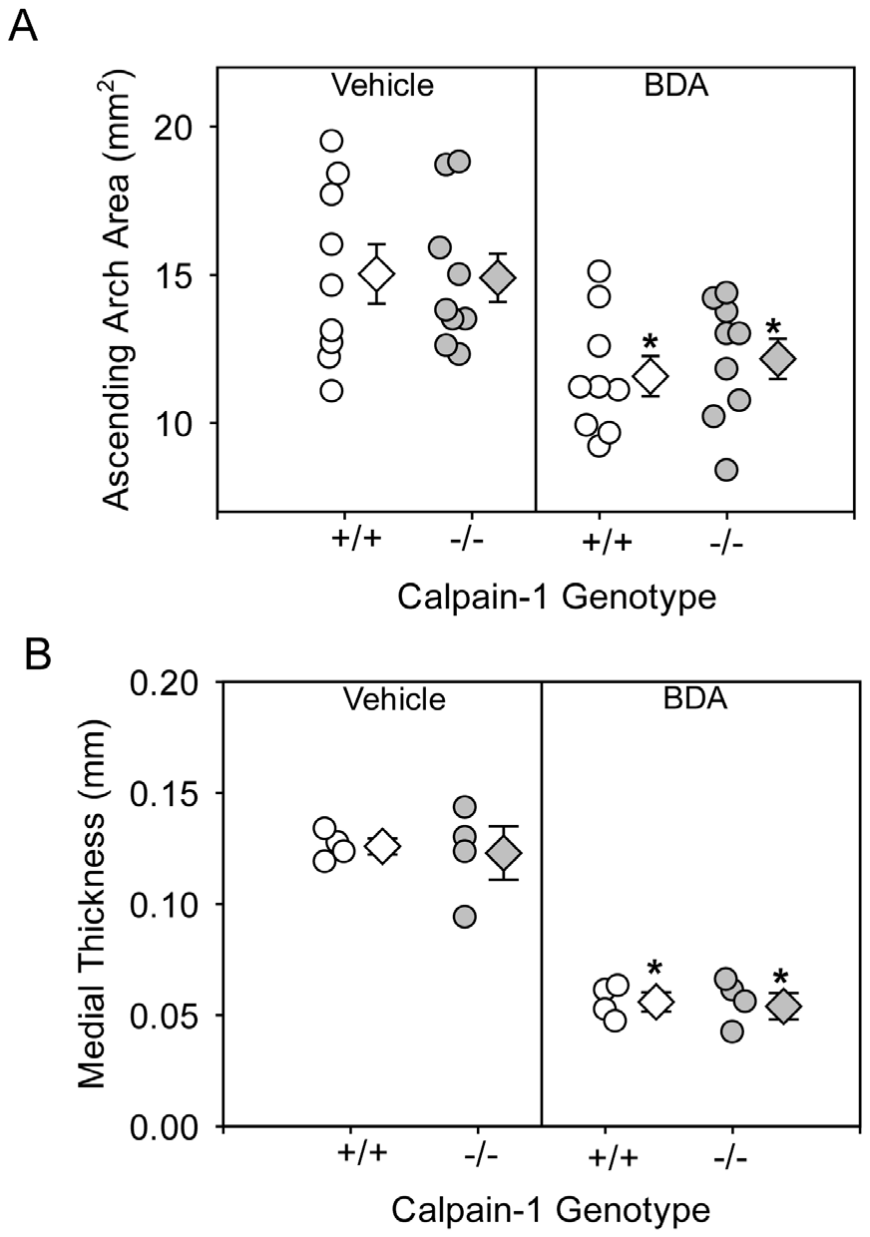
BDA-410 administration attenuated AngII-induced ascending aorta dilation and aortic medial thickness in calpain-1 deficient mice. **A.** Measurement of intimal areas of ascending aortas on day 28 (n = 10–12). **B.** Measurement of medial thickness measured from internal to external lamina (n = 10–12). Open circles (calpain-1 +/+) and gray circles (calpain-1 −/−) represent individual mice, diamonds represent means, and bars are SEMs. Statistical analyses were performed using Two way ANOVA with a Holm-Sidak multiple comparison post-hoc test. * denotes *P*<0.05 when comparing vehicle versus BDA-410.

**Figure 10:**
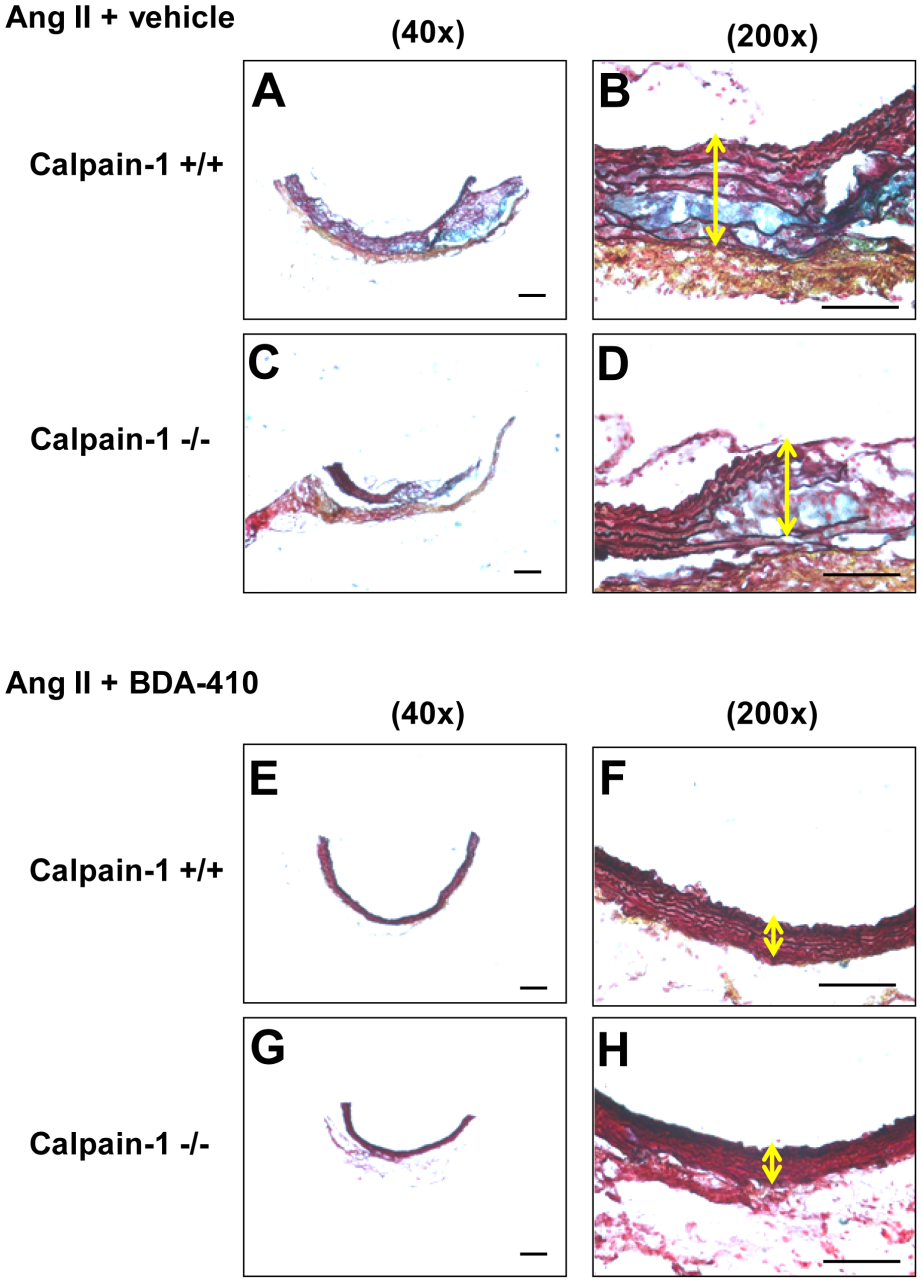
BDA-410 administration suppressed ascending aortic medial break in calpain-1 +/+ and −/− mice. Representative anterior ascending aortic tissue sections from AngII+vehicle (**A–D**) and AngII+BDA-410 (**E–H)** administered calpain-1 +/+ and calpain-1 −/− mice stained with Movat’s pentachrome. Arrow indicates medial break. Scale bars correspond to 50 µm. A,C,E and G = 40×; B,D,F and H = 200×.

**Figure 11:**
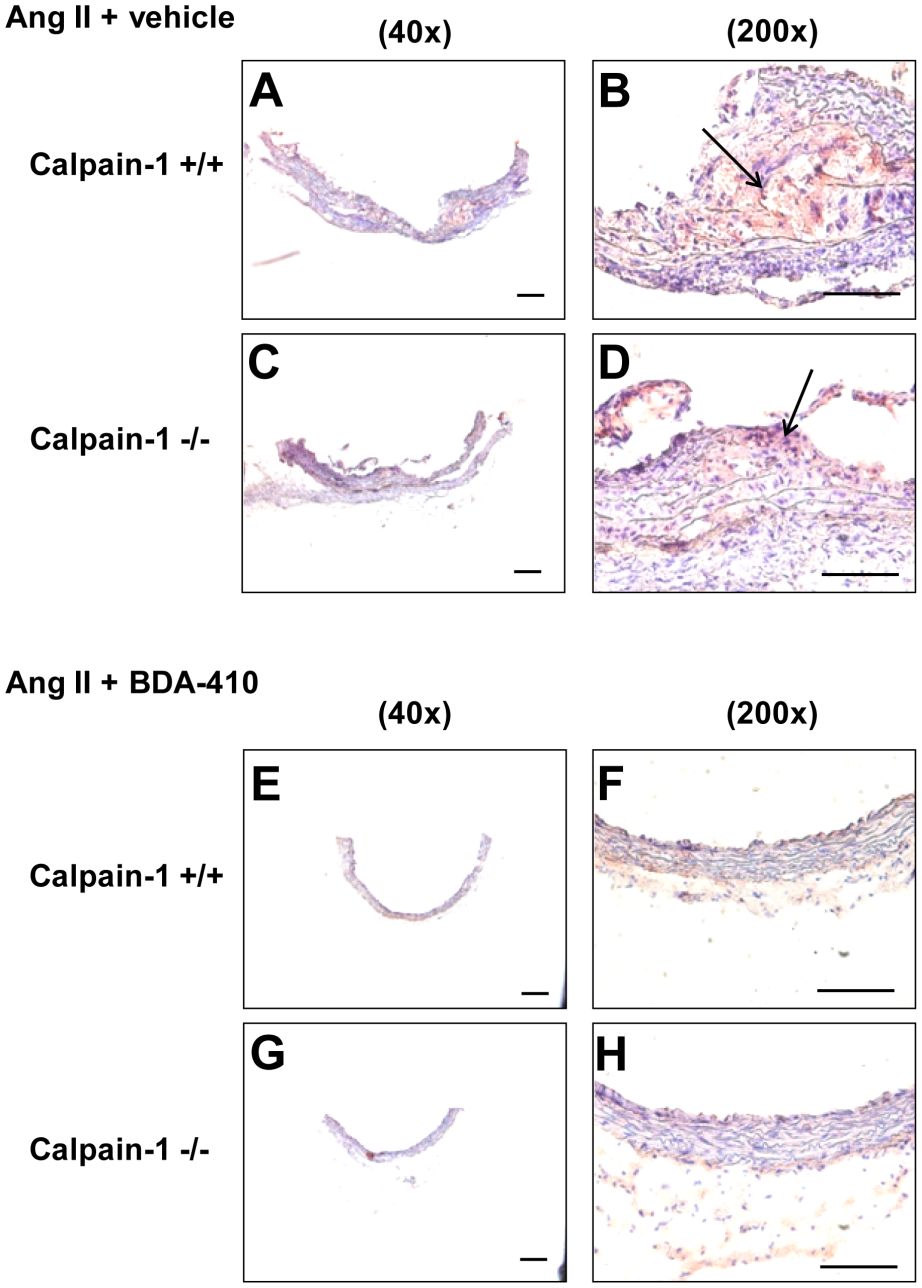
BDA-410 administration reduced macrophage accumulation in ascending aortas of calpain-1 +/+ and −/− mice. Representative anterior ascending aortic tissue sections from AngII+vehicle (**A–D**) and AngII+BDA-410 (**E–H)** administered calpain-1 +/+ and calpain-1 −/− mice immunostained for CD68 (**E–H)**. CD68+ cells stain red. Arrow indicates positive staining with CD68. Scale bars correspond to 50 µm. A,C,E and G = 40×; B,D,F and H = 200×.

Next we examined the effect of BDA-410 administration on AngII-induced ascending AA formation in both calpain-1 +/+ and −/− mice. Dilation of ascending aortic intimal area was measured as an index of severity of aneurysm. AngII infusion increased ascending aortic area in vehicle-administered calpain-1 +/+ and −/− mice to a similar extent (Fig. 9A). In contrast, administration of BDA-410 significantly attenuated AngII-induced ascending aorta dilation in both calpain-1 +/+ and −/− groups (*P*<0.05; Fig. 9A). In addition, AngII infusion increased ascending aortic medial thickness (Fig. 9B) as measured from inner to outer elastic lamina. As reported earlier, Movat’s pentachrome staining showed that increased aortic medial thickness is associated with an expansion of intraelastic spaces towards the adventitial aspect of the media (Fig. 10 A–D). Movat’s staining also showed that AngII-induced medial thickness in the intra-lamellar medial space was associated with the deposition of proteoglycans and glycosaminoglycans (Fig. 10 A–D). Further, immunostaining for macrophages using a CD68 antibody, showed the accumulation of CD68+ macrophages in the expanded intra-lamellar medial space (Fig. 11 A–D). Administration of BDA-410 significantly attenuated AngII-induced medial thickness (*P*<0.05; Fig. 9B), extracellular matrix and macrophage accumulation in the ascending aortas of both calpain-1 +/+ and −/− mice (Fig. 10 E–H, Fig. 11 E–H).

### Calpain Inhibition Attenuated AngII-induced Filamin-A Breakdown in the Aorta

**Figure 12:**
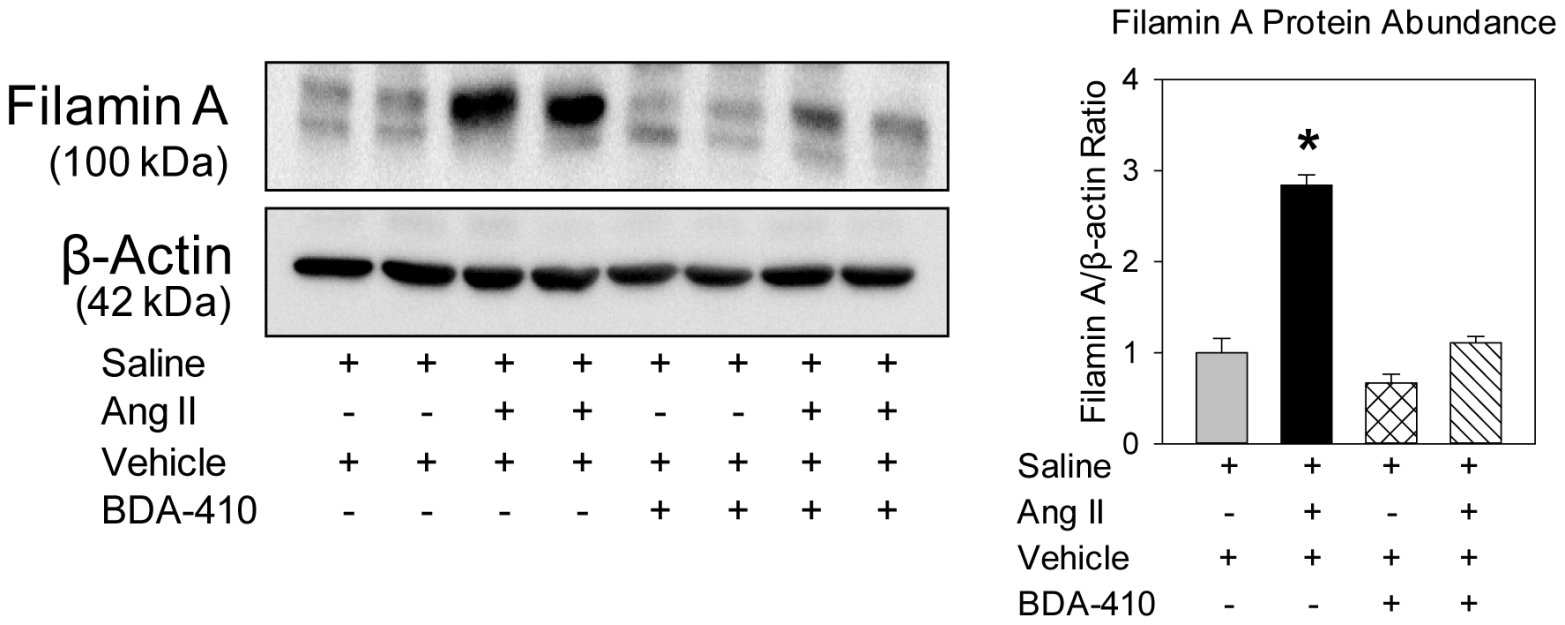
BDA-410 administration attenuated AngII-induced C-terminal fragmentation of filamin A in aortas of LDL receptor −/− mice. Filamin A protein was detected by Western blotting in aortic tissue extracts from saline and AngII infused mice administered either vehicle or BDA-410 (n = 4). β-actin was shown as the loading control. Results are represented as means ± SEMs; Statistical analyses were performed using two-way ANOVA with a Holm-Sidak multiple comparison post-hoc test. * represent significance of *P*<0.05 when vehicle compared to BDA-410.

A recent proteomic analysis study using aortic medial tissue protein extract from Marfan Syndrome patients showed upregulation of the C-terminal fragmentation of filamin A, that was highly correlated with elevated calpain-2 activity [31]. Filamin A, an actin binding protein, is known to be required for cell-cell contact during vascular development. Filamin A protein also contributes to organization and stability of the actin cytoskeleton, integrates cellular signaling cascades, and regulates cellular functions including adhesion and motility [32]. Therefore, we examined whether AngII infusion increases the C-terminal fragmentation of filamin A in the aorta and whether BDA-410 administration had any influence on this process. Male LDL receptor −/− mice fed a high fat-enriched diet were infused with either saline or AngII for 14 days. The calpain inhibitor, BDA-410, was administered at a dose of 30 mg/kg per day by gavage one week before infusion and throughout the subsequent 14 days. AngII infusion significantly increased C-terminal fragmentation of filamin A as analyzed by Western blotting using an antibody specific for the C-terminal domain of filamin A (*P*<0.05; Fig. 12). In contrast, BDA-410 administration along with AngII significantly blunted AngII-induced filamin A fragmentation in the aortas (*P*<0.05; Fig. 12).

## Discussion

In the present study, we examined the role of calpain-1 in AngII-induced ascending and abdominal AAs. Here, we demonstrated that calpain-1 deficiency had no discernible effects on AngII-induced ascending and abdominal AAs. Administration of a calpain specific inhibitor, BDA-410, significantly attenuated AngII-induced ascending and abdominal AA formation in both calpain-1 +/+ and −/− mice. The beneficial effect of calpain inhibition was associated with the reduction of macrophage accumulation, and blunted the AngII-induced C-terminal fragmentation of actin binding protein, filamin A, in the aorta.

Calpain-1 deficiency did not show any effects on AngII-induced blood pressure elevation. Consistent with the current observation, our earlier study of calpain inhibition using BDA-410 also showed no effect on AngII-induced increased blood pressure [14]. This result is in agreement with an earlier study in which overexpression of calpastatin attenuated AngII-induced cardiac hypertrophy without affecting blood pressure [33]. Further, development of AngII-induced abdominal or ascending AAs is shown to be independent of increases in blood pressure [34], [10].

In the present study, whole body deficiency of calpain-1 had no influence on size and incidence of either AngII-induced abdominal or ascending AAs. However, administration of calpain inhibitor, BDA-410, significantly attenuated development of AngII-induced vascular pathologies. These findings indicate that complete inhibition of calpain activity is required to attenuate AngII-induced AAs. An interesting finding here is the observation that during AngII infusion, calpain-1 deficiency is associated with an increase in calpain-2 protein abundance. In addition, elevated level of calpain-2 contributed to total aortic calpain activity in calpain-1 deficient mice compared to calpain-1 +/+ aortas, as evident by increased breakdown of its substrate protein, spectrin, and also by increased hydrolysis of fluorescent labeled calpain substrate. AngII infusion into wild type mice had no effect on calpain-2 protein abundance in the aorta, which is consistent with our earlier findings showing that AngII infusion into hypercholesterolemic mice increased aortic calpain-1, but not calpain-2, protein and activity [14]. These observations clearly demonstrate that there is a compensatory increase of calpain-2 by AngII in calpain-1 deficient mice to maintain the total aortic calpain activity. However, it is not clear whether the two calpain isoforms, -1 and -2, are acting synergistically or independently in mediating development of AngII-induced AAs. Given the fact that calpain-1 deficiency had no effect on AngII-induced aortic pathologies, depletion of calpain-2 itself may be sufficient to attenuate development of AngII-induced AAs. However, embryonic lethality of calpain-2 deficiency in mice impedes development of whole body calpain-2 deficient mice. Recently, calpain-2 floxed mice have been developed, that will serve as a unique tool to study the role of cell specific calpain-2 in development of AngII-induced AAs [35]. In support of a specific role of calpain-2 in development of ascending AAs, a proteomics study of aortic media in patients with Marfan syndrome revealed a higher level of calpain-2 protein and calpain activity [31]. Increased calpain activity was positively correlated with fragmentation of actin binding protein filamin A in the dilated aortic media [31].

Macrophages are one of the major leukocytic components present in both AngII-induced ascending and abdominal AAs [10], [11]. Similar to our earlier studies [14] and other published reports, [11] AngII promoted medial macrophage accumulation in the abdominal aortas. In contrast, ascending aortas from calpain-1 +/+ and −/− mice showed considerable accumulation of macrophages throughout the intralamellar spaces on the adventitial site of the vessel. In agreement, previous studies also demonstrated increased macrophage accumulation on the adventitial side of the ascending aorta with AngII infusion [12], [10]. Consistent with our previous study, BDA-410 administration attenuated macrophage accumulation in abdominal aortas as well as ascending aortas of both groups of mice. The beneficial effect observed with calpain inhibition on initial macrophage accumulation raises a question of possible involvement of leukocytic-derived calpains in development of AngII-induced vascular pathologies. However, bone marrow transplantation studies using AT1a receptor deficient mice failed to demonstrate a role of bone marrow-derived AT1a receptors in the development of AngII-induced ascending and abdominal AAs [12], [13]. In fact, these studies suggested involvement of AT1a receptors present in the resident vessel wall cells. With respect to ascending AAs, depletion of AT1a receptors in endothelial cells, not in smooth muscle cells, partially reduced ascending aortic dilation [12]. Also deficiency of CCR2, the cognate receptor of MCP-1, suppressed AngII-induced ascending aortic dilation to a similar extent as observed with endothelial AT1a receptor deficient mice [10]. Based on these studies, calpain-derived from aortic endothelial cells may be involved in the breakdown of aortic medial tissue by enhancing the C-terminal fragmentation of actin binding protein filamin A and thereby promoting ascending aorta dilation.

In support of this hypothesis, proteomics analysis of aortic media protein from Marfan syndrome patients showed increased fragmentation of filamin A protein with positive correlation of increased calpain activity [31]. Although filamin A can be cleaved by caspase activity as well as granzyme B, proteomics analysis showed no alterations in activity of either caspase or granzyme B in the aortic media of Marfan syndrome patients [31]. This observation further supports the potential effect of calpain activity on filamin A degradation in Marfan patients. In a published abstract, Kim and Dietz groups have reported a recurrent mis-sense mutation at the calpain cleavage site of filamin A in patients with ascending AA. Furthermore, they also demonstrated that in cells isolated from ascending AA patients, calpain inhibition blocked transforming growth factor-β induced epithelial to mesenchymal transition, thus preserving epithelial cell morphology [36]. In our present study, we also observed an increased C-terminal fragmentation of filamin A with AngII-infusion in aortas. In support to the above mentioned clinical reports, administration of calpain inhibitor, BDA-410, completely attenuated the C-terminal fragmentation of filamin A by AngII. Altogether, these findings strongly support a possible role of increased calpain activity in filamin A degradation in development of AngII-induced ascending AA.

In summary, using calpain-1 deficient mice and a novel calpain inhibitor, BDA-410, we demonstrate a functional role of calpain activity in development of AngII-induced ascending and abdominal AAs. Inhibition of calpain activity may offer a new therapeutic target to prevent aortic dilation in patients with Marfan syndrome and abdominal AA. However, further studies are warranted to evaluate the specific role of calpain-2 and the cellular sources of calpain activity in development of AngII-induced AAs. The recent availability of calpain-2 floxed mice will be helpful to identify the source and functional role of calpain-2 in future studies.

## Supporting Information

File S1 **File includes Figures S1–S6.** Figure S1. Genotyping of experimental mice for calpain-1 alleles by PCR. Genomic DNA from tail biopsies was isolated and screened by PCR for calpain-1 wild type (+/+) and null (−/−) alleles. Reaction products were sized using agarose gel electrophoresis. Figure S2. Calpain-1 deficiency did not affect lipoprotein cholesterol distributions. Lipoproteins were resolved by size-exclusion chromatography. Total cholesterol concentrations are expressed as mean absorbance per fraction. Symbols represent the means and bars are SEMs of 5 individual mice per group: Calpain-1 +/+ (circles) and calpain-1 −/− (triangles). Figure S3. Examples of vascular pathology measurements. Ultrasound images (A - Day 0 and Day 28), ex vivo pictures of suprarenal aortas (B - after termination) and ascending arch aortas (C), that represent aortic diameters nearest the mean of each group. Figure S4. Calpain-1 deficiency had no effect on AngII-induced atherosclerosis in LDL receptor −/− mice. Atherosclerotic lesion areas were measured on aortic arch intimal surfaces (n = 18–19). Open circles (calpain-1 +/+) and gray circles (calpain-1 −/−) represent individual mice, diamonds represent means, and bars are SEMs. Statistical analyses were performed with nonparametric Mann-Whitney Rank sum test. Figure S5. Histological and cellular characteristics of AngII-induced abdominal AAs in calpain-1 +/+ and calpain-1 −/− mice. Representative suprarenal aortic tissue-sections from AngII infused calpain-1 +/+ and calpain-1 −/− mice stained with Movat’s pentachrome (A–D) and immunostained for CD68 (E–H). Elastin stains black; CD68+ cells stain red. Scale bars corresponds to 50 µm. Arrow indicates medial break and positive staining with CD68. A,C,E and G = 40×; B,D,F and H = 200×. Figure S6. Histological and cellular characteristics of AngII-induced ascending AAs in calpain-1 +/+ and calpain-1 −/− mice. Representative anterior ascending aortic tissue-sections from AngII infused calpain-1 +/+ and calpain-1 −/− mice stained with Movat’s pentachrome (A–D) and immunostained for CD68 (E–H), Elastin stains black; CD68+ cells stain red. Scale bars corresponds to 50 µm. Arrow indicates medial break and positive staining with CD68. A,C,E and G = 40×; B,D,F and H = 200×.
(PDF)
