# Supplementary material for: Calpain-2 Compensation Promotes Angiotensin II-Induced Ascending and Abdominal Aortic Aneurysms in Calpain-1 Deficient Mice
Source: PLoS One. 2013 Aug 19;8(8):e72214. doi: 10.1371/journal.pone.0072214 (PMC3747148; doi:10.1371/journal.pone.0072214)
Supplement: File S1 — File includes Figures S1–S6. Figure S1. Genotyping of experimental mice for calpain-1 alleles by PCR. Genomic DNA from tail biopsies was isolated and screened by PCR for calpain-1 wild type (+/+) and null (−/−) alleles. Reaction products were sized using agarose gel electrophoresis. Figure S2. Calpain-1 deficiency did not affect lipoprotein cholesterol distributions. Lipoproteins were resolved by size-exclusion chromatography. Total cholesterol concentrations are expressed as mean absorbance per fraction. Symbols represent the means and bars are SEMs of 5 individual mice per group: Calpain-1 +/+ (circles) and calpain-1 −/− (triangles). Figure S3. Examples of vascular pathology measurements. Ultrasound images (A - Day 0 and Day 28), ex vivo pictures of suprarenal aortas (B - after termination) and ascending arch aortas (C), that represent aortic diameters nearest the mean of each group. Figure S4. Calpain-1 deficiency had no effect on AngII-induced atherosclerosis in LDL receptor −/− mice. Atherosclerotic lesion areas were measured on aortic arch intimal surfaces (n = 18–19). Open circles (calpain-1 +/+) and gray circles (calpain-1 −/−) represent individual mice, diamonds represent means, and bars are SEMs. Statistical analyses were performed with nonparametric Mann-Whitney Rank sum test. Figure S5. Histological and cellular characteristics of AngII-induced abdominal AAs in calpain-1 +/+ and calpain-1 −/− mice. Representative suprarenal aortic tissue-sections from AngII infused calpain-1 +/+ and calpain-1 −/− mice stained with Movat’s pentachrome (A–D) and immunostained for CD68 (E–H). Elastin stains black; CD68+ cells stain red. Scale bars corresponds to 50 µm. Arrow indicates medial break and positive staining with CD68. A,C,E and G = 40×; B,D,F and H = 200×. Figure S6. Histological and cellular characteristics of AngII-induced ascending AAs in calpain-1 +/+ and calpain-1 −/− mice. Representative anterior ascending aortic tissue-sections from AngII infused calpain- [file pone.0072214.s001.pdf]

Figure S1

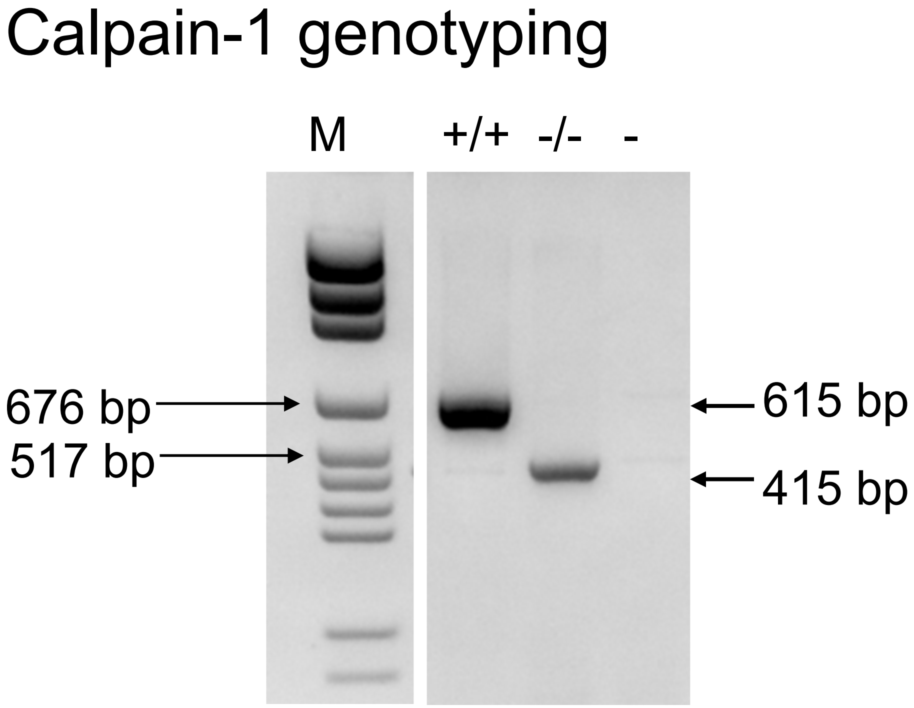

Figure S1. Genotyping of experimental mice for calpain-1 alleles by PCR. Genomic DNA from tail biopsies was isolated and screened by PCR for calpain-1 wild type (+/+) and null (-/-) alleles. Reaction products were sized using agarose gel electrophoresis.

Figure S2

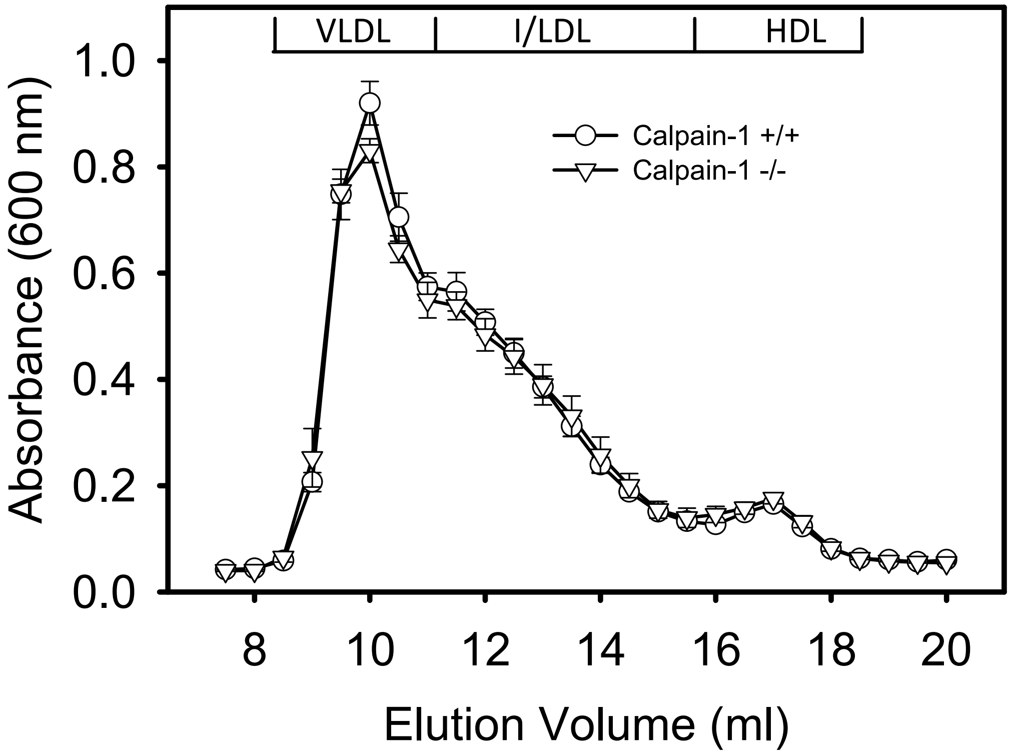

Figure S2. Calpain-1 deficiency did not affect lipoprotein cholesterol distributions. Lipoproteins were resolved by size-exclusion chromatography. Total cholesterol concentrations are expressed as mean absorbance per fraction. Symbols represent the means and bars are SEMs of 5 individual mice per group: Calpain-1 +/+ (circles) and calpain-1 -/- (triangles).

Figure S3

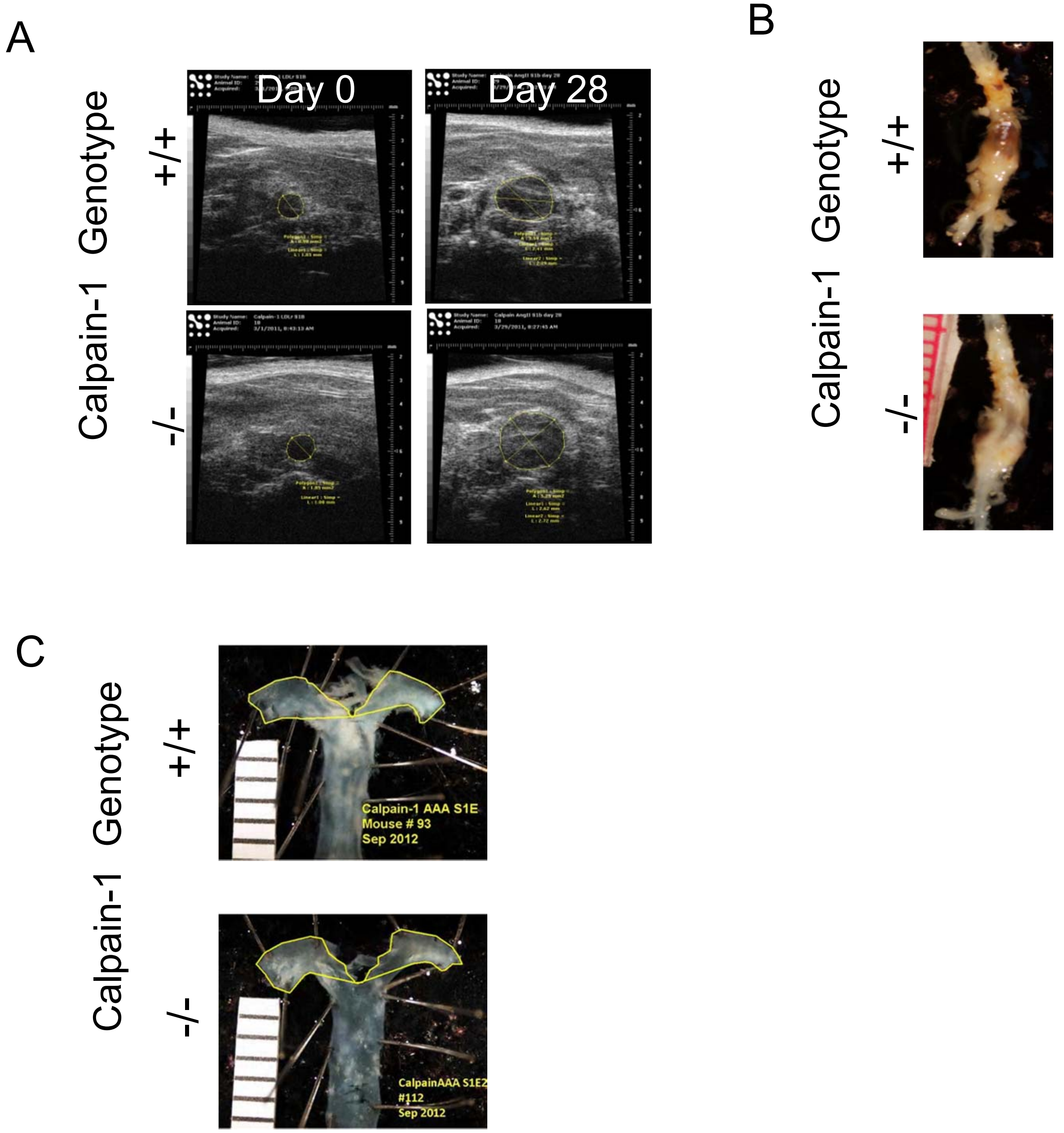

Figure S3. Examples of vascular pathology measurements. Ultrasound images (**A** - Day 0 and Day 28), ex vivo pictures of suprarenal aortas (**B** - after termination) and ascending arch aortas (**C**), that represent aortic diameters nearest the mean of each group.

Figure S4

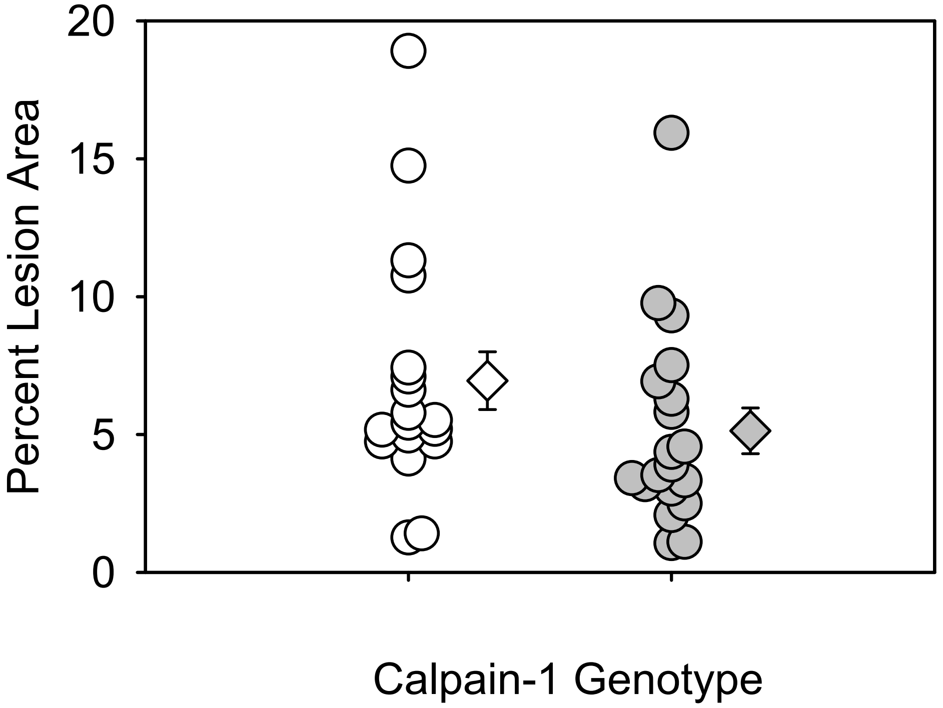

Figure S4. Calpain-1 deficiency had no effect on AngII-induced atherosclerosis in LDL receptor -/- mice. Atherosclerotic lesion areas were measured on aortic arch intimal surfaces (n = 18-19). Open circles (calpain-1 +/+) and gray circles (calpain-1 -/-) represent individual mice, diamonds represent means, and bars are SEMs. Statistical analyses were performed with nonparametric Mann-Whitney Rank sum test.

Figure S5

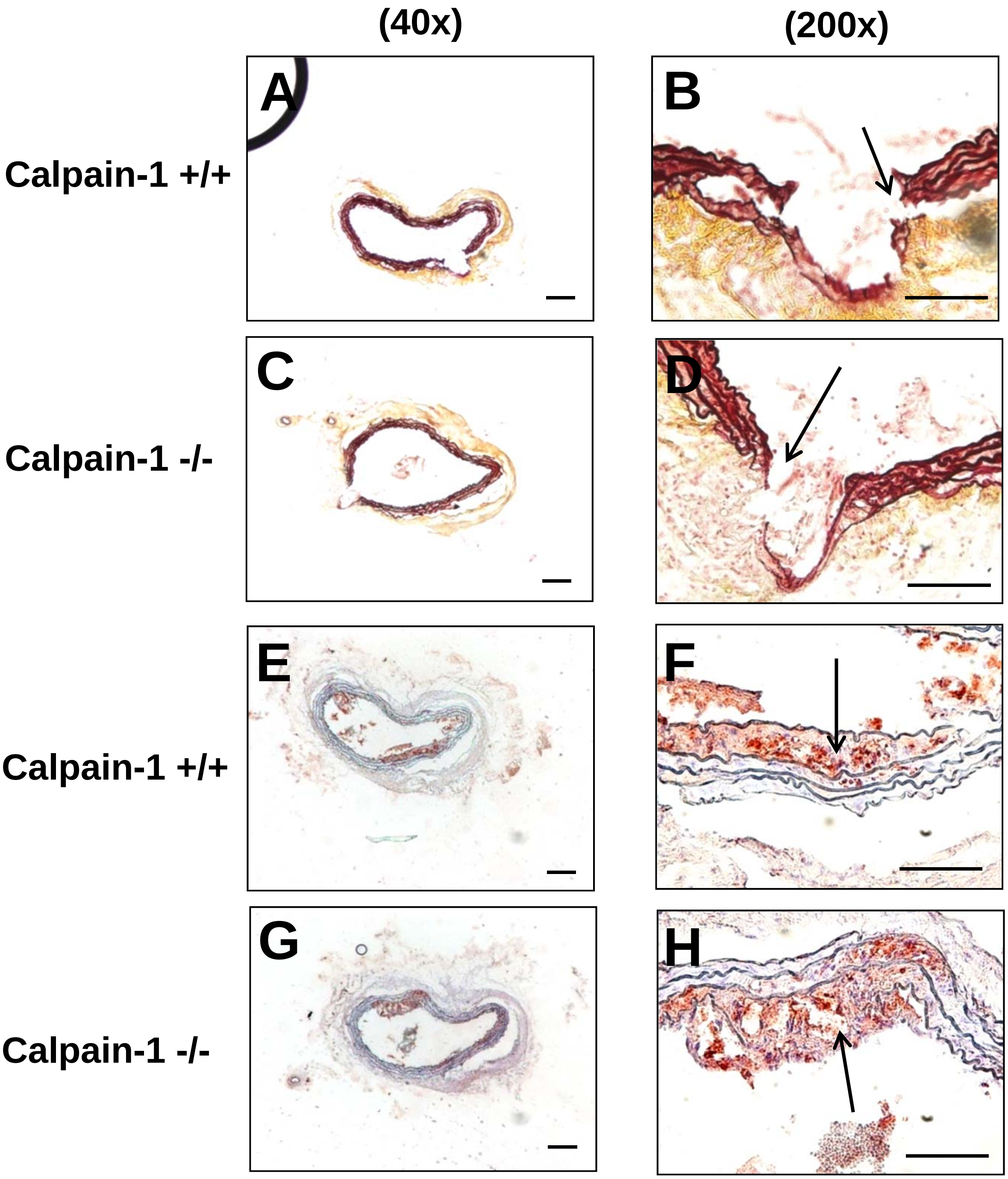

Figure S5. Histological and cellular characteristics of AngII-induced abdominal AAs in calpain-1 +/+ and calpain-1 -/- mice. Representative suprarenal aortic tissue-sections from AngII infused calpain-1 +/+ and calpain-1 -/- mice stained with Movat's pentachrome (**A-D**) and immunostained for CD68 (**E-H).** Elastin stains black; CD68+ cells stain red. Scale bars corresponds to 50µm. Arrow indicates medial break and positive staining with CD68. A,C,E and G = 40x; B,D,F and H = 200x.

Figure S6

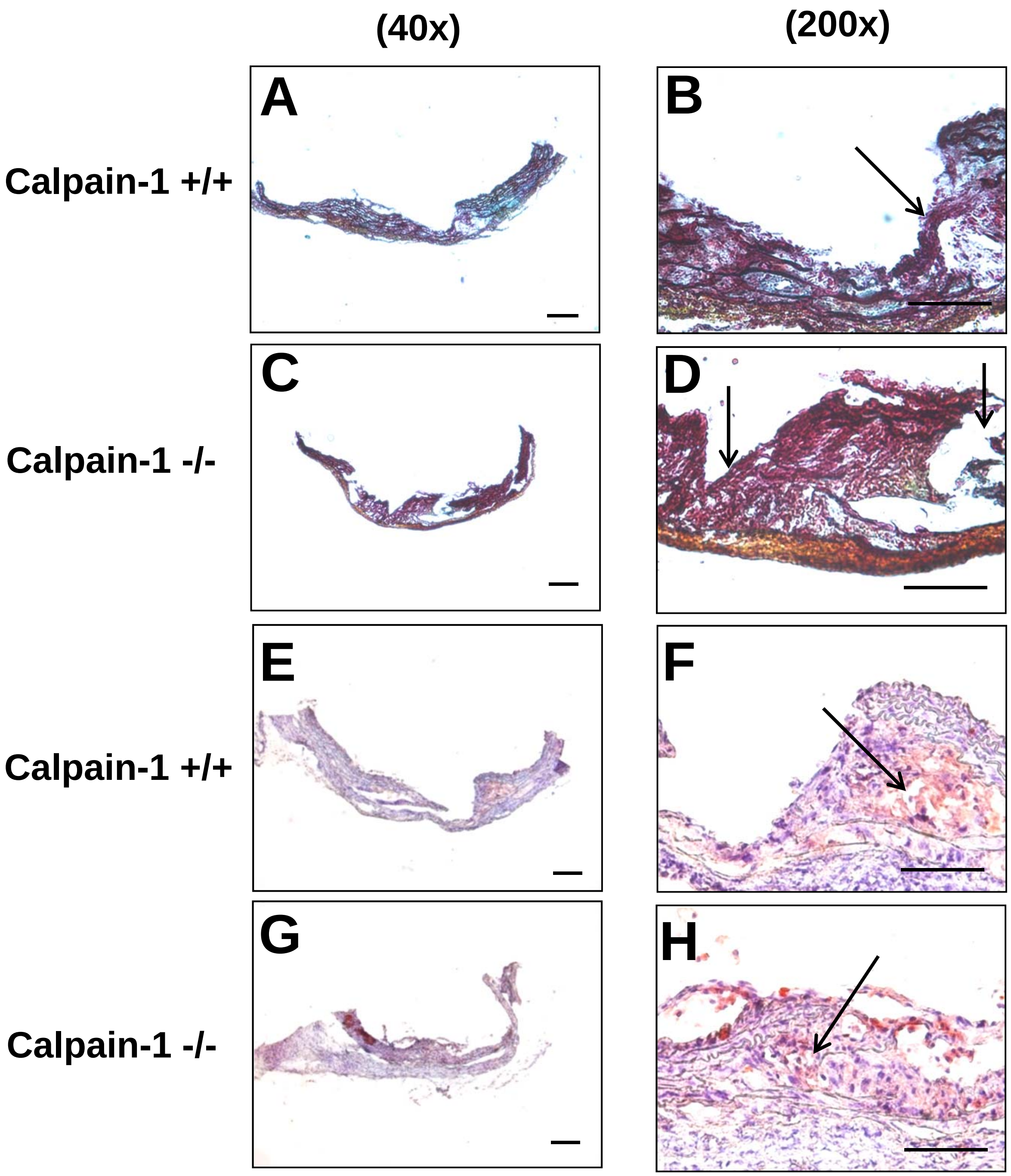

Figure S6. Histological and cellular characteristics of AngII-induced ascending AAs in calpain-1 +/+ and calpain-1 -/- mice. Representative anterior ascending aortic tissue-sections from AngII infused calpain-1 +/+ and calpain-1 -/- mice stained with Movat's pentachrome (**A-D**) and immunostained for CD68 (**E-H)**, Elastin stains black; CD68+ cells stain red. Scale bars corresponds to 50μm. Arrow indicates medial break and positive staining with CD68. A,C,E and G = 40x; B,D,F and H = 200x.
